# The effect of beta-blockers on hemodynamic parameters in patient-specific blood flow simulations of type-B aortic dissection: a virtual study

**DOI:** 10.1038/s41598-021-95315-w

**Published:** 2021-08-06

**Authors:** Mohammad Amin Abazari, Deniz Rafiei, M. Soltani, Mona Alimohammadi

**Affiliations:** 1 Department of Mechanical Engineering, K. N. Toosi University of Technology, Tehran, Iran; 2 Department of Electrical and Computer Engineering, Faculty of Engineering, School of Optometry and Vision Science, Faculty of Science, University of Waterloo, Waterloo, Canada; 3 Advanced Bio Initiative Center, Multidisciplinary International Complex, K. N. Toosi University of Technology, Tehran, Iran; 4 Centre for Biotechnology and Bioengineering (CBB), University of Waterloo, Waterloo, ON Canada; 5 Cancer Biology Research Center, Cancer Institute of Iran, Tehran University of Medical Sciences, Tehran, Iran

**Keywords:** Biomedical engineering, Mechanical engineering, Aortic diseases

## Abstract

Aortic dissection (AD) is one of the fatal and complex conditions. Since there is a lack of a specific treatment guideline for type-B AD, a better understanding of patient-specific hemodynamics and therapy outcomes can potentially control the progression of the disease and aid in the clinical decision-making process. In this work, a patient-specific geometry of type-B AD is reconstructed from computed tomography images, and a numerical simulation using personalised computational fluid dynamics (CFD) with three-element Windkessel model boundary condition at each outlet is implemented. According to the physiological response of beta-blockers to the reduction of left ventricular contractions, three case studies with different heart rates are created. Several hemodynamic features, including time-averaged wall shear stress (TAWSS), highly oscillatory, low magnitude shear (HOLMES), and flow pattern are investigated and compared between each case. Results show that decreasing TAWSS, which is caused by the reduction of the velocity gradient, prevents vessel wall at entry tear from rupture. Additionally, with the increase in HOLMES value at distal false lumen, calcification and plaque formation in the moderate and regular-heart rate cases are successfully controlled. This work demonstrates how CFD methods with non-invasive hemodynamic metrics can be developed to predict the hemodynamic changes before medication or other invasive operations. These consequences can be a powerful framework for clinicians and surgical communities to improve their diagnostic and pre-procedural planning.

## Introduction

Cardiovascular disease is the first most common cause of death worldwide^1,2^. Aortic dissection (AD) is a subset of cardiovascular disease, which occurs when an injury to the intima layer enables blood to enter the middle layer of the aortic wall. It leads to create a channel by communicating the main bloodstream or true lumen (TL) of the aorta into a false lumen (FL), which is separated by an intimal flap (IF). This can happen in either the ascending or descending aorta, for which the disease is classified into Stanford type-A or Stanford type-B AD, respectively^3–5^.

Although prompt surgical intervention is the recommended treatment method for type-A AD, there is no specific treatment guideline for type-B AD. This is partially due to a lack of knowledge of the biomechanical and hemodynamic properties of AD^6,7^. The ideal treatment of type-B AD for each patient can be determined by compromising the cost and impact of each treatment scenario and specific conditions of the patients^5,8,9^, which is known as patient-specific therapy^3,10,11^. Two main reasons for the progression of type-B AD are high blood flow and elevated blood pressure (BP)^11,12^. Type-B AD patients should initially receive medical treatment to control and decrease the BP, heart rate^5,11^, and wall shear stress (WSS) forces^13^. Beta-blocker (BB) family groups are the initial routine pharmacological therapy of type-B AD^14,15^. The physiological responses of BB decrease left ventricular contractions by affecting the level of ions^16–19^. This mechanism leads to the reduction of aortic BP and heart rate to lower than 120 mmHg and 60 BPM, respectively^15–17^; for additional BP reduction and in the presence of life-threatening conditions like malperfusion in branch vessels or vessel wall’s rupture, surgical operation or thoracic endovascular aortic repair (TEVAR) is essentially needed^8,20–22^. However, the rate of reoperation for patients treating with surgery is about 10% higher than medical intervention after about 12 years^10,12^. Furthermore, underlying and high-risk conditions such as stroke and early mortality may prioritize surgical treatment over pharmacological therapy^23^. In acute AD patients, the primary purposes of pharmacological treatment are to stabilise the dissection, accelerate healing, prevent rupture, and reduce the risk of complications^15^. A better understanding of the disease's progression and therapy response virtually before the operation can help clinical researchers comprehend the pathophysiology of AD and, finally, appropriate the optimal solution.

In recent years, several studies have reported the importance of personalised computational fluid dynamics (CFD) methods along with imaging techniques to develop diagnosis and controlling approaches of aortic disease, in order to customize patient-specific treatment outcomes^23–29^. Some studies have shown the importance of the combination of particle image velocimetry and CFD simulations in type-B AD in pre-procedural planning, clinical decision support, and CFD models validation^30,31^. Several studies have focused on TEVAR based on the prediction of thrombus formation and growth of the FL^13,32–35^. Recent work has demonstrated the potential use of CFD simulations in the hemodynamic analysis of type-B AD before and after treatment with multilayer flow modulators stents^36^. Advances in the investigation of hemodynamic metrics have allowed for the diagnosing of common pathologies in type-B AD patients, which have aided clinicians in their decision-making process. For example, an elevated time average wall shear stress (TAWSS) index plays a key role in the vessel wall’s rupture^4,37^. Furthermore, highly oscillatory, low magnitude shear (HOLMES) has shown promising results in predicting the calcified regions^6^. Additionally, analysis of pressure values can be helpful in the study of aneurysmal dilatation and malperfusion^23,38^. A more recent study has shown the importance of TAWSS and pressure difference values between the TL and FL in the prediction of rapid aneurysmal expansion in the dissected aorta^39^, confirming the need for further measuring of hemodynamic metrics for the prediction of further progression and development of AD. Furthermore, a recent review has indicated the importance of CFD tools and hemodynamic features for risk assessment in AD patients, which can be used by clinicians^40^. Moreover, boundary condition accuracy in CFD simulations of AD has been improved by replacing flow split boundary and constant pressure with physiologically accurate dynamic three-element Windkessel (WK3)^3,41^. Deploying WK3 for the outlets of AD simulations is particularly important as the correct calculation of flow (at the outlets), which is crucial in case of malperfusion (lack of blood supply)^6^. What is more, the CFD methods with patient-specific data have shown promising results in hemodynamic changes^41–43^ and prediction of hemodynamic outcomes in virtual stenting operations^23^. For instance, Xu et al. have recently introduced metrics by using computational hemodynamic analysis on 51 patients with type-B AD to evaluate the hemodynamic improvement, which can be used to predict luminal remodeling of patients after TEVAR^44^. In contrast, no single study has been reported to quantify the variation in hemodynamics during medical treatment in a clinical case of type-B AD coupled with the WK3. The present study aims to highlight the effectiveness of using the pharmaceutical treatment on type-B AD in terms of progression, planning, and management.

In the current study, a numerical blood flow simulation in a patient-specific geometry of type-B AD coupled with the WK3 model is created for three different heart rates undergoing virtual medication. Non-invasive hemodynamic metrics that influence disease progression are investigated and compared in each case. The primary goal of the present work is to represent a framework to better understand and predict how the heart rate reduction mechanism of anti-hypertensive drugs with changing the hemodynamics tend to control the progression and development of the disease, which can be investigated from WSS results. Toward this goal, clinicians and surgical communities can choose the optimal patient-specific solution regarding the risk of other treatment methods (TEVAR or open surgery) and the investigated biomechanical medication metrics. Additionally, since the present model is used for CFD simulation on a patient-specific level, different treatment scenarios can be applied to each patient prior to any medical or surgical intervention. The present model will be one more step forward for breaking down barriers to patient-specific AD therapy.

## Material and methods

### Geometry and grid generation

The three-dimensional (3D) fluid domain is generated from a stack of 887 digital imaging and communications in medicine (DICOM) images of a 58-year-old male patient suffering from Stanford type-B AD. The fluid domain is reconstructed using MIMICS Research 21.0 (2018 release, Materialise, Leuven, Belgium). The resolution of the computed tomography (CT) images is 0.5 mm/pixel, IF thickness is about 1.5–2.5 mm between the two tears, and the diameter of the ascending aorta (D) is approximately 4.5 cm. Several processes are used to create different masks to fully capture the geometry.

**Figure 1:**
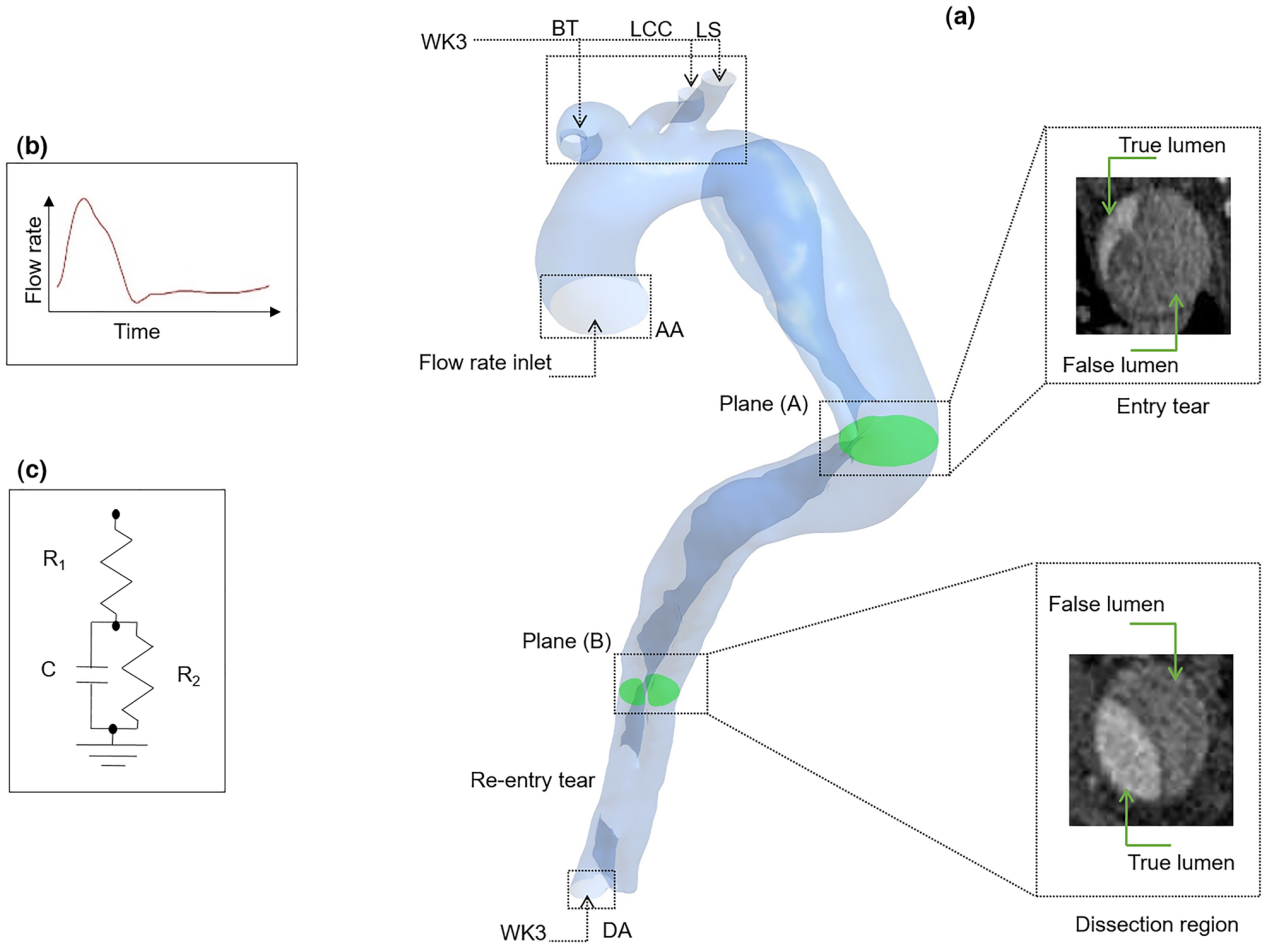
Multi-scale patient-specific model domain and its boundary conditions. (**a**) Patient-specific dissected aorta geometry with two planes to illustrate the dissection region and entry tear. Boundaries are named with green arrows; *DA* descending aorta, *LS* left subclavian artery, *AA* ascending aorta, *LCC* left common carotid artery, *BT* brachiocephalic trunk (**b**) Inlet flow rate diagram. (**c**) Three-element Windkessel (WK3) boundary condition.

The three-branch arteries (i.e., brachiocephalic artery, subclavian artery, and left carotid artery) are retained on the aortic arch; however, other small vascular branches are excluded due to the low quality of the images. Additionally, the iliac arteries are omitted, and finally, boundaries are cut with parallel planes in order to place the inlet and outlets along the same axis. As shown in Fig. 1, two cross-sectional planes of the reconstructed 3D geometry are mapped back to CT images to better understand the multi-scale patient-specific model domain.

The fluid domain is meshed using ANSYS Meshing 18.2 (ANSYS Inc., Canonsburg, PA, USA). The geometry consists of about 420,000 and 175,000 tetrahedral cells and node numbers, respectively. To minimise the computational errors near the wall, seven prismatic layers with a growth rate of 1.2 are deployed. Mesh independence is performed for the fluid domain; a coarse mesh with about 250,000 cells and a fine mesh with about 1,000,000 cells are created to evaluate the grid independence. The pressure values in all three cases are evaluated so that there is a maximum of 3.5% difference between the coarse and medium grids, and a 0.7% of maximum difference between the medium and fine grids. In order to save computer costs, the medium mesh is chosen.

### Computational fluid dynamics

The continuity and Navier-Stokes equations (Eqs. (1) and (2), respectively) are solved by CFD software ANSYS-CFX 18.2 (ANSYS Inc., Canonsburg, PA, USA) based on the finite volume method. ANSYS CFD-Post and MATLAB (R2018b version, Mathworks, Natick, USA) are used to Post-processing the data. Discretization of the governing equations involves a second-order backward Euler scheme with a time step of 0.005 s, and maximum residual mean square errors are set to 1×10^−5^:

1 $$ \nabla \cdot \vec{u} = 0 $$

2 $$ \rho \frac{{\partial {\vec{\text{u}}}}}{\partial t} + \rho \left( {{\vec{\text{u}}} \cdot \nabla } \right){\vec{\text{u}}} + \nabla p - \mu \Delta \vec{u} = 0 $$

where $\vec{u}$ is the fluid velocity vector and *μ*, $\rho$, and $p$ represent the local dynamic viscosity, density, and pressure, respectively.

### Boundary conditions

The blood is assumed as an incompressible fluid with a density^3^ of 1056 kg/m^3^. The fluid is considered as a non-Newtonian model, with viscosity determined by the Carreau–Yasuda viscosity model, wherein *μ* is viscosity, *γ*′ is shear rate, *μ*_0_ is Carreau–Yasuda zero shear viscosity and *μ*_*∞*_, *a*, *m*, and *λ*_*CY*_ are Carreau–Yasuda infinite shear viscosity, Yasuda exponent, Carreau–Yasuda Power Law Index, and Carreau–Yasuda time constant, respectively:

3 $$ \mu = (\mu_{0} - \mu_{\infty } )(1 + (\lambda_{CY} \gamma^{\prime})^{a} )^{(m - 1)/a} + \mu_{\infty } $$

The parameters used in the present work are determined by Gijsen et al.^45^, which are shown in Table 1.

**Table 1:** Parameters used for the Carreau–Yasuda model^45^.

*μ*_0_ [m pa s]	*μ*_*∞*_ [m pa s]	*a*	*m*	*λ*_*CY*_ [s]
22	2.2	0.644	0.392	0.110

In the present study, the case with the highest velocity (86 BPM, 0.69 s for one cardiac cycle, frequency (*f*) = 1.43 Hz)^46^ is used to calculate the Reynolds number. The critical Reynolds number in the aorta has been determined by Stalder et al.^47^:

4 $$ Re_{c} = 169\alpha^{0.83} St^{ - 0.27} $$

5 $$ \alpha = 0.5D\sqrt {2\pi f\rho \mu^{ - 1} } $$

6 $$ St = 0.5\;fD(V_{p} - V_{m} )^{ - 1} $$

where *Re*_*c*_, *α*, and *St* are the critical Reynolds, Womersley, and Strouhal numbers, respectively. *V*_*m*_ and *V*_*p*_ are the mean and peak velocities, which are about 0.05 m/s and 0.28 m/s, respectively. The Womersley number in AA is about 34.7, the Strouhal number is 0.14, the mean and peak Reynolds numbers are approximately 640 and 3400, respectively, and the critical Reynolds number is about 5500. Since the peak Reynolds number is lower than the critical Reynolds number, the inlet flow is assumed to be subcritical during the cycle, so a laminar flow model is used. This usually occurs in large arteries due to the low mean flow velocity^3,13,48,49^.

**Figure 2:**
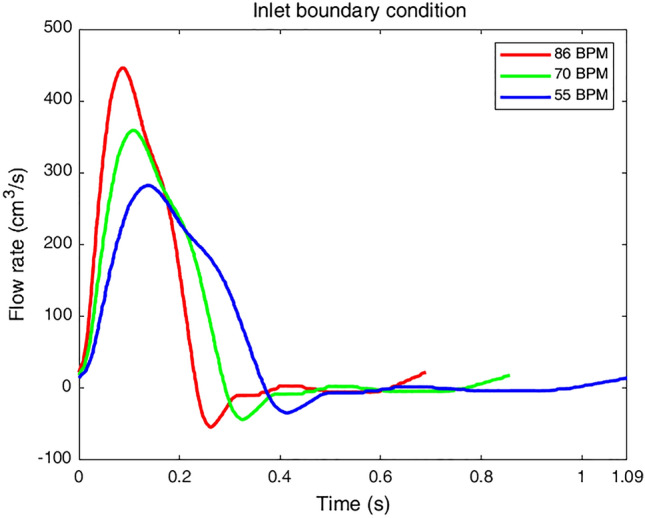
Input flow waves at the ascending aorta for all three cases.

As discussed in the introduction, anti-impulse therapy decreases patient’s heart rate to the normal range^8,20,21,50^. For this purpose, the patient-specific model is used to provide three case studies with different heart rates to investigate the virtual pharmacological treatment outcomes. In the present study, it is assumed that the patient's heart rate drops from 86 BPM (high-heart rate) to 70 BPM (moderate-heart rate) and, finally, to 55 BPM (regular-heart rate)^23,46^. Three aforementioned virtually adjusted input flow rate diagrams are shown in Fig. 2.

Since the inlet flow to the AA is not available for this patient, Karmonik et al*.*'s results^46^ are used and adjusted accordingly^23^. Three cardiac cycles are applied to reach periodic repetitive states, and the last one is used to extract all the results.

This study is carried out in order to provide a framework for clinicians to better tune their patient-specific pharmacological treatment. Therefore, computational cost (simulation time) is of great importance herein. On the other hand, the inclusion of vessel wall simulation–fluid–solid interaction (FSI)—would require great resources and adequate time^13,27,37^. Additionally, setting up FSI simulation is not clinician-friendly; thus rigid wall with no-slip boundary condition is assumed.

A three-element Windkessel model is applied to each outlet to achieve realistic dynamic boundary conditions. This model uses a hydraulic-electrical analogy (0-D), in which pressure (*P*) and blood flow (*Q*) represent voltage and current in a circuit, respectively (see Fig. 1c)^3^:

7 $$ P = (R_{1} + R_{2} )Q - R_{2} C\frac{dP}{{dt}} + R_{1} R_{2} C \frac{dQ}{{dt}} $$

where *R*_1_, *R*_2_, and *C* are characteristic impedance, hydrodynamic resistance, and compliance, respectively. Equation (8) shows the discretized form of the above equation using the backward Euler method, which is defined in ANSYS-CFX via CFX Expression Language (CEL)^3,6^:

8 $$ P_{n} = \frac{{\left( {R_{1} + R_{2} + R_{1} \beta } \right)Q_{n} - R_{1} \beta Q_{n - 1} + \beta P_{n - 1} }}{1 + \beta } $$

where *n* and *n* − 1 are the current and previous time steps and *Δt* in $\beta = \frac{{R_{2} C}}{\Delta t}$ is the time step. All the parameters are taken from a similar study^51^.

The last step in implementing the accurate boundary condition is changing the resistance properties of the WK3 model at different physiological states (high, moderate, and regular-heart rate), which is related to the pharmacological effects of the conventional BBs^16–18,52^. Thus, five different sets of peripheral resistance in the WK3 model are assumed. The resistance values at high-heart rate are decreased by 20 and 25% and in the moderate case, these values are decreased by 10 and 15%. It should be noted that all of these reductions are calculated based on the resistance values at the regular-heart rate^51^, and the changes in resistance values are accounted to all outlet boundary conditions (BT, LCC, LS, and DA). These thresholds in the resistance values are selected based on the previous works^53–55^. Taylor et al. have suggested that the total microvascular resistance at hyperemic condition reduces up to 24% of the healthy value^53,55^. Recently, Randles et al. have used a 25% reduction in microvascular resistance at hyperemic condition compared with the normal one^54^.

In the present study, a sensitivity analysis to investigate the effect of different peripheral resistance on hemodynamic metrics is performed. The input parameters of the sensitivity analysis are five different boundary conditions at three different heart rates (20 and 25% reduction in resistance at 86 BPM, 10 and 15% reduction in resistance at 70 BPM and the based WK3 parameters at 55 BPM). The average pressure along with the aorta, TAWSS and HOLMES distributions, the percentage difference in TAWSS, OSI and HOLMES metrics are considered as the key hemodynamic output parameters. It should be noted that the velocity distribution and flow patterns in all the cases during the sensitivity analysis are also examined and shown the same trends.

## Results

### Velocity distribution

**Figure 3:**
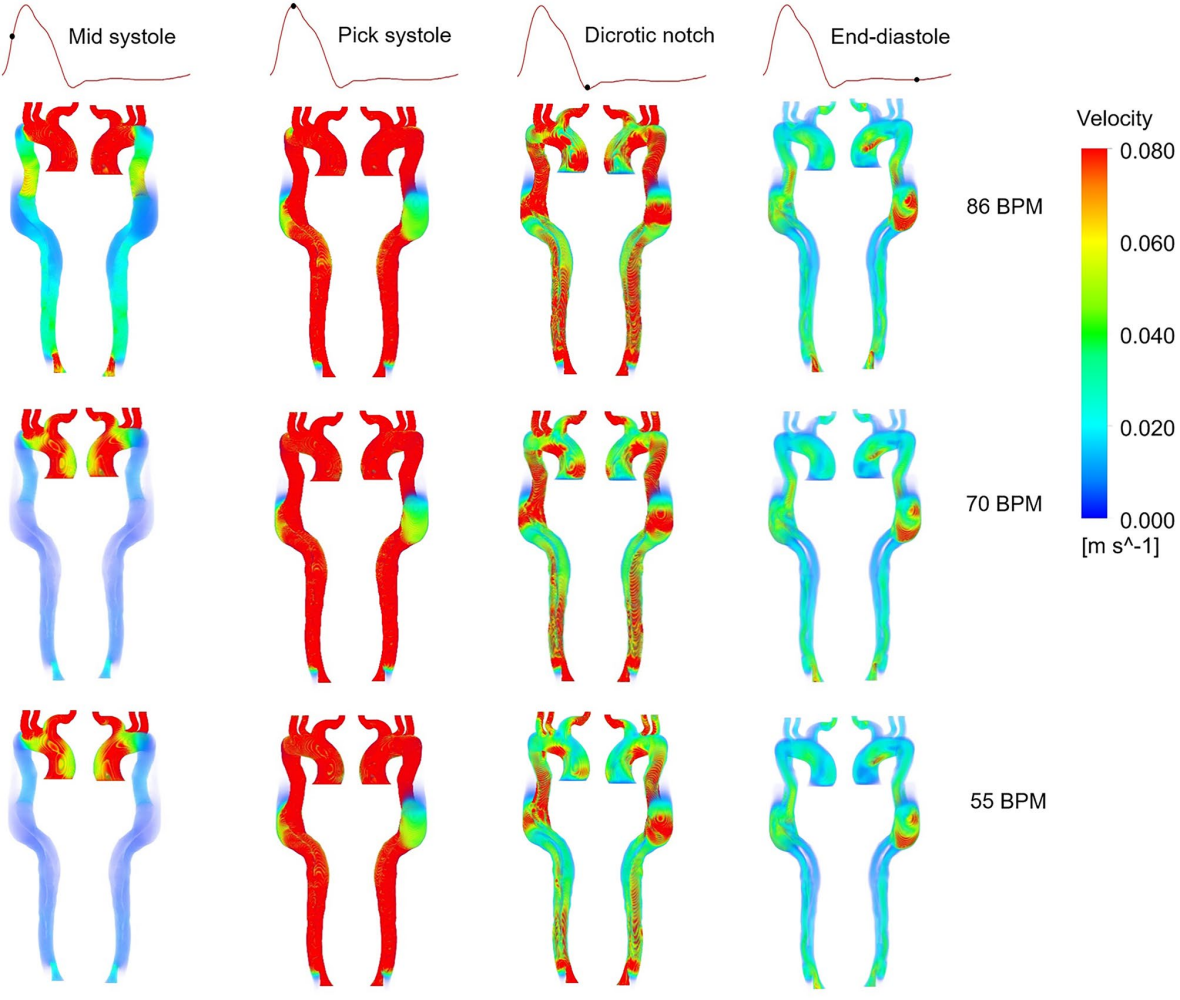
Velocity distribution for all three cases at four cardiac points (the scale bar shown in the figure indicates all contours).

**Figure 4:**
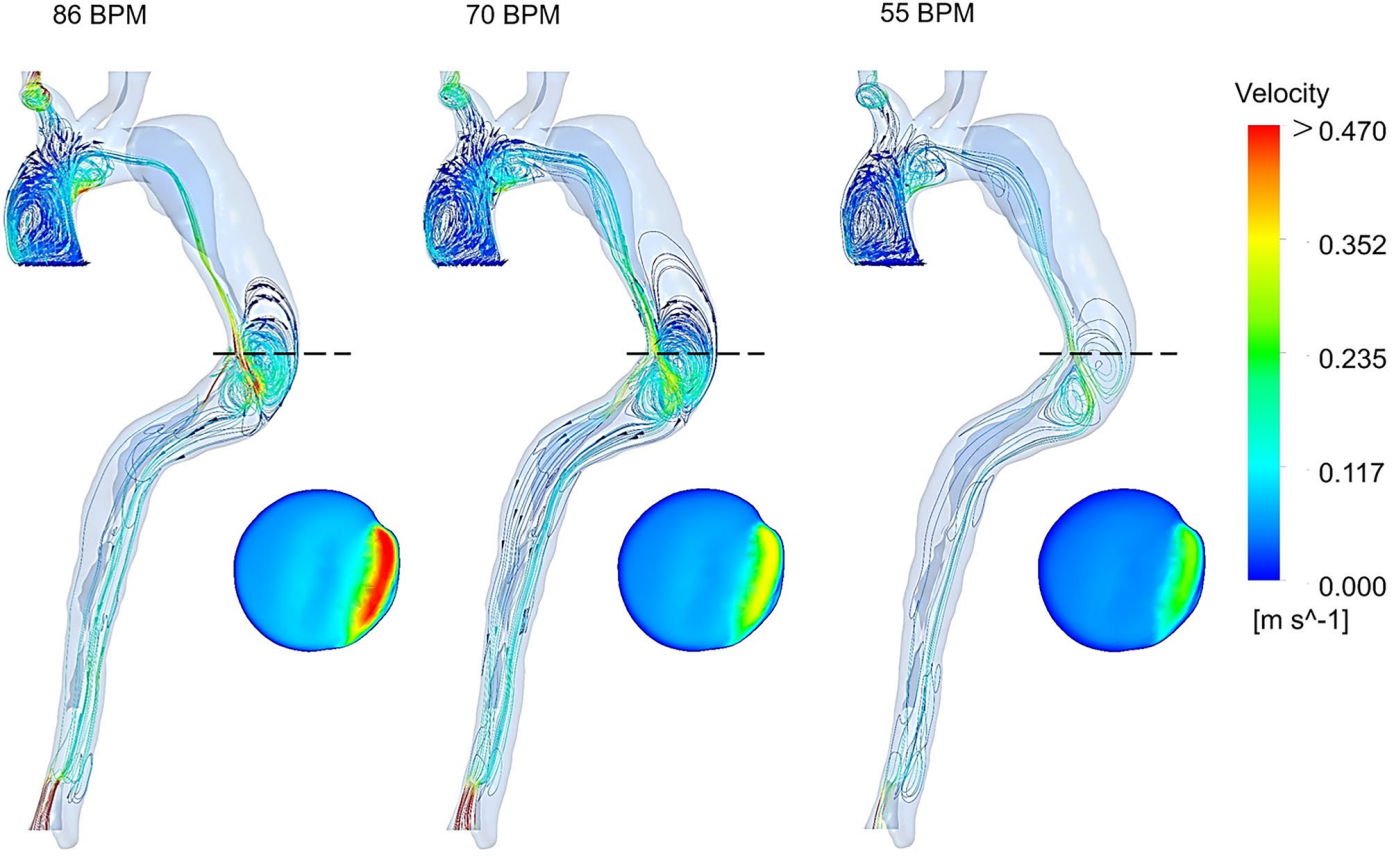
Streamlines and velocity contours during end-diastole (dashed lines indicate the position of selected cross-sectional planes in the middle of entry tear and the scale bar shown in the figure indicates all contours).

The velocity distribution in three cases is shown in Fig. 3. Generally, the velocity decreases with the heart rate reduction at all cardiac points except pick systole, where no significant changes can be seen. Velocity magnitude in the upper branches remains the same in three cases, whereas decreasing heart rate is led to a more uniform gradient in the descending aorta of moderate and regular-heart rate cases than the high-heart rate case. These changes are more visible in the WSS indicators’ results, which are discussed further in the following section. Figure 4 shows streamlines during end-diastole in three cases. At this stage in the cardiac cycle, most of the blood flows through the BT, while a small proportion of the total aortic flow enters through the FL by entry tear. A few of these streamlines, which have higher velocity, wash the FL wall and make the helical structures around the entry tear. The length of these complex flows, their number, and velocity values decrease during anti-impulse therapy, which can be observed in velocity contours at the cross-sectional planes in the middle of entry tear in Fig. 4.

### Pressure distribution

**Figure 5:**
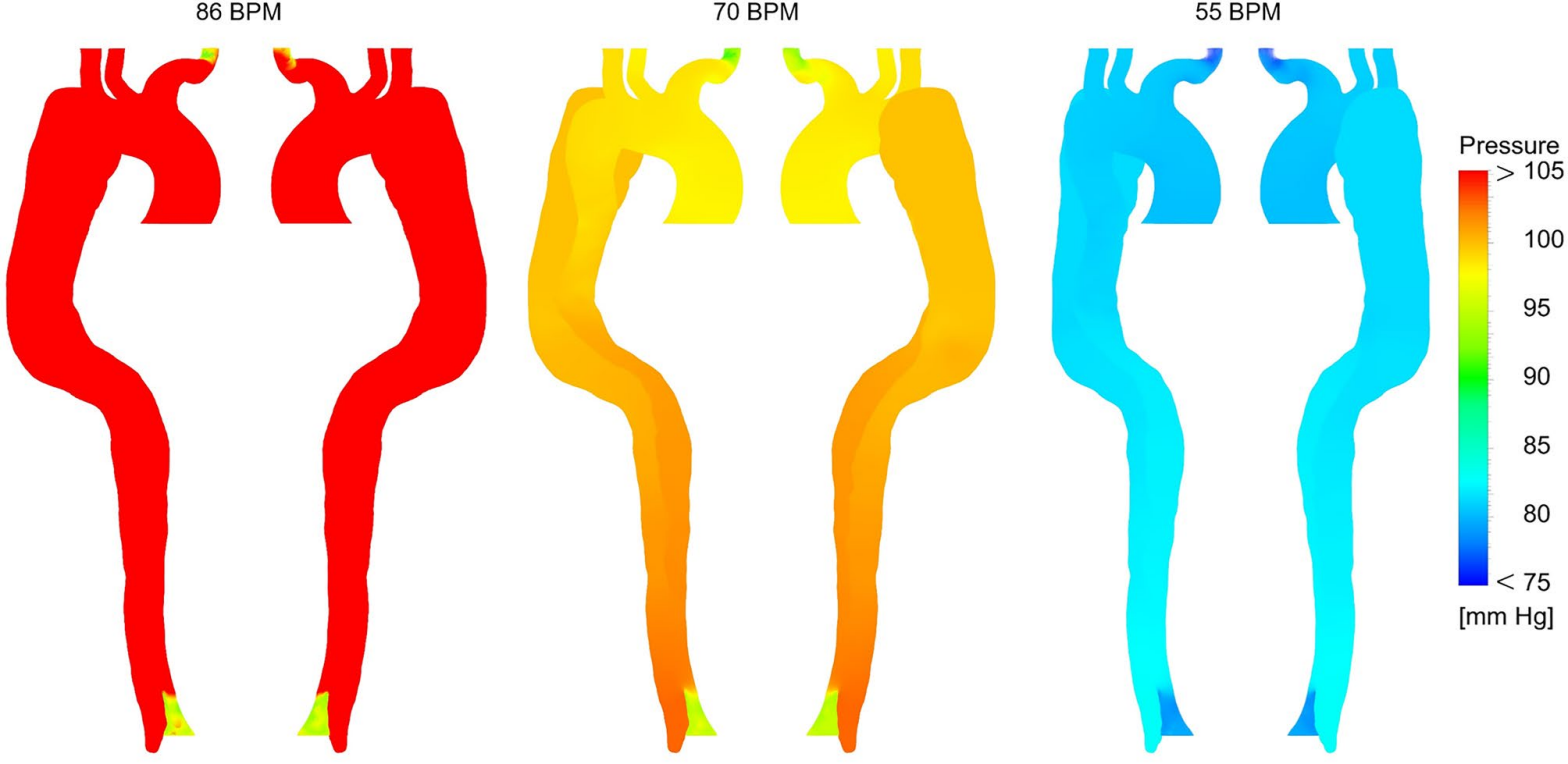
Pressure distribution for three cases during pick systolic blood pressure (the scale bar shown in the figure indicates all contours).

The pressure distribution along all the cases during pick systolic BP is shown in Fig. 5. The sensitivity analysis for pressure results at 86 and 70 BPM with different reductions in resistance values can be found in Supplementary Table 1 and Supplementary Table 2, respectively. These results show the percentage difference in pressure values during pick systolic BP at 86 and 70 BPM are lower than 2%. Thus, the pressure distributions in Fig. 5 are shown only for the case with a 20% reduction in resistance at 86 BPM and the case with a 15% reduction in resistance at 70 BPM.

Results show a significant difference in pressure values between different heartbeats. Although pressure in each case has a fairly uniform gradient throughout the domain, the average pressure is notably decreased from about 112 mmHg at 86 BPM to approximately 100 and 85 mmHg at 70 and 55 BPM, respectively. In the high-heart rate case, the pressure difference between AA and DA is about 18 mmHg (from about 112 mmHg is dropped to about 94 mmHg), implying a severe hypertension situation. On the other hand, the average pressure drop decreases to 12 and 7 mmHg in moderate and regular-heart rate cases, respectively. Additionally, pressure magnitude at BT is between 100 and 95 mmHg without considerable change in high and moderate-heart rate cases.

### WSS indicators

**Figure 6:**
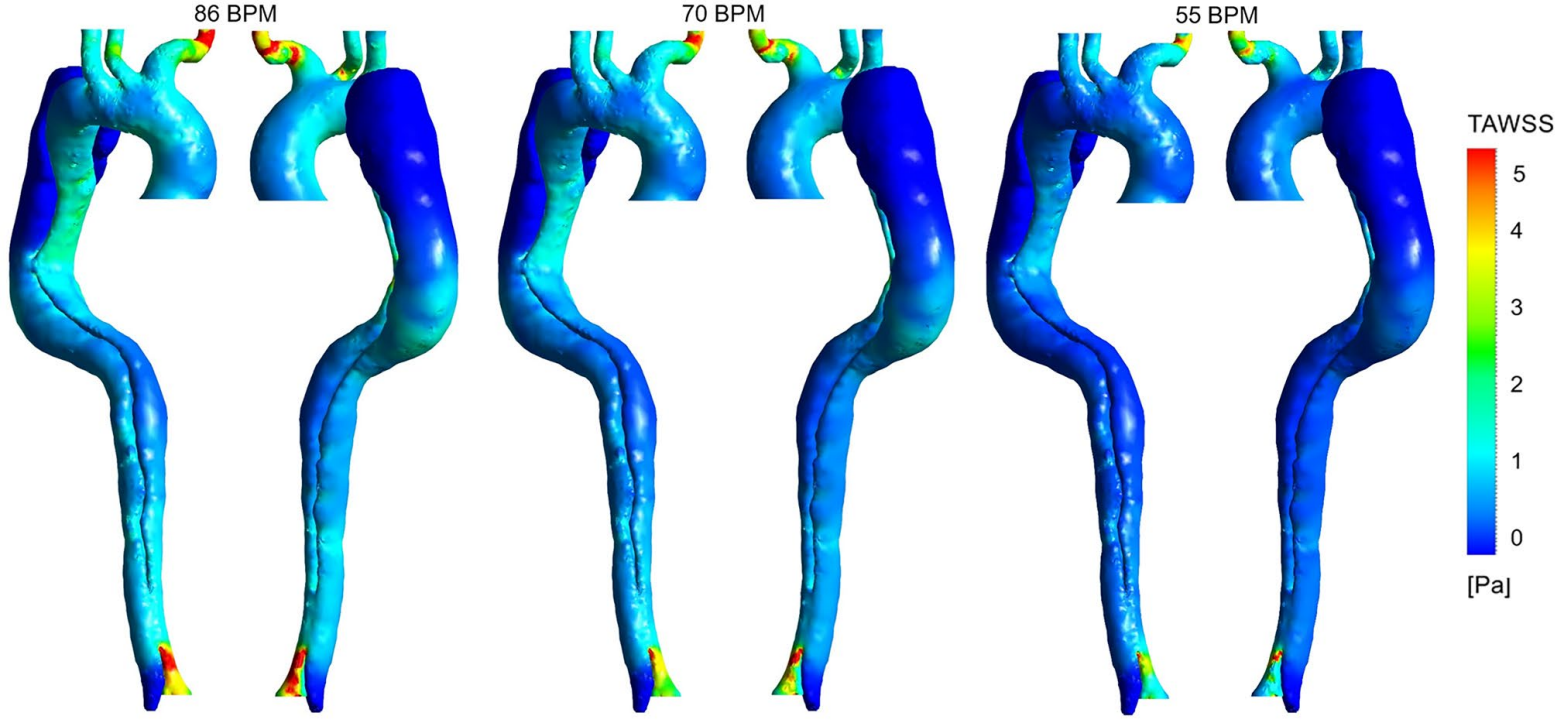
Time-averaged wall shear stress (TAWSS) distribution for three cases (the scale bar shown in the figure indicates all contours).

**Figure 7:**
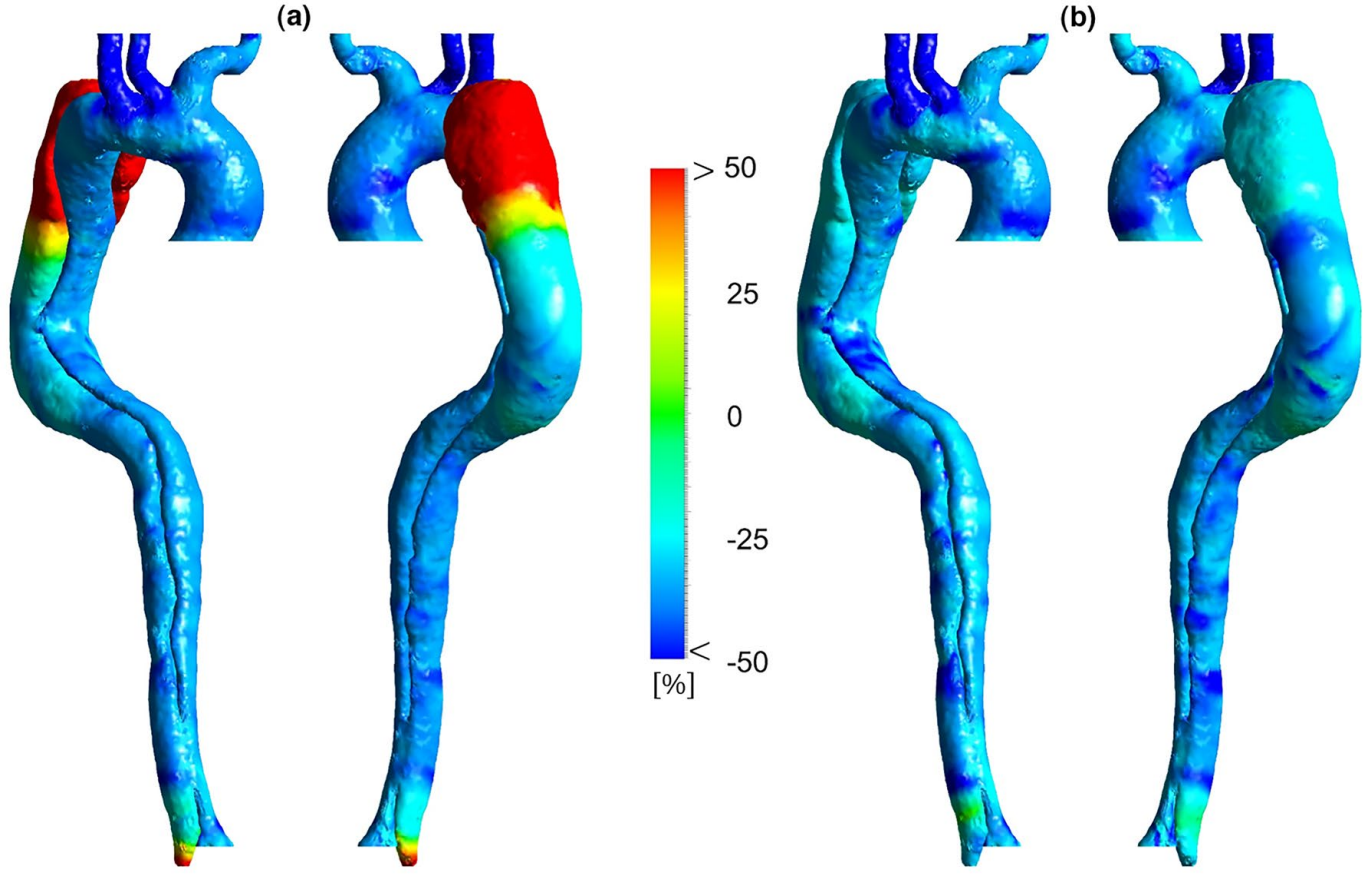
Percentage difference in time-averaged wall shear stress (TAWSS) according to different heart rates. (**a**) Percentage difference in TAWSS between 86 and 70 BPM. (**b**) Percentage difference in TAWSS between 55 and 70 BPM.

TAWSS is an important WSS indicator which is prescribed by Eq. (9)^3^:

9 $$ TAWSS = \frac{1}{T}\int_{0}^{T} {\left| {\vec{\tau }\left( t \right)} \right| dt} $$

where $\vec{\tau }(t)$ and *T* are WSS vector at time *t* and the total time of the cardiac cycle, respectively. This equation is adjusted to all 3D geometry nodes to evaluate TAWSS. TAWSS distribution for each case and the percentage difference between each two are shown in Figs. 6 and 7, respectively.

Sensitivity analysis for TAWSS distribution at 86 and 70 BPM with different reductions in resistance values is performed, the results of which are shown in Supplementary Figures 1a, 2a, 3, and 4. Supplementary Figures 3 and 4 indicate that the TAWSS distribution along the aorta for different resistance values has the same trend at each heart rate. According to Supplementary Figures 1a and 2a, the average percentage difference in TAWSS along the aorta at 86 and 70 BPM are 0.25 and 0.42%, respectively. In other words, at most parts of the aorta, the percentage difference in TAWSS varies between ± 1%, except in some regions at the outer surface of FL after the aortic arch. Although, the absolute TAWSS values in these regions have remained without considerable changes. It should be noted that TAWSS results in Figs. 6 and 7 are shown only for the case with a 25% reduction in resistance at 86 BPM and the case with a 10% reduction in resistance at 70 BPM.

Figure 6 indicates that the highest values of TAWSS are in the upper branches (especially BT) and around DA. TAWSS value at AA and TL around entry tear fluctuates between 2 and 2.5 Pa in the high-heart rate model. This value at TL decreases to 1–1.5 Pa and 0.5–1 Pa in moderate and regular models, respectively. The reduction of velocity gradient leads to the development of a low TAWSS value at the outer surface of TL around the entry tear to both downstream and upstream directions (see Fig. 3). Eventually, in the regular model, TAWSS in distal FL and TL is almost zero. Very low TAWSS value and slight velocity gradient in the distal FL around DA indicate no way out for blood flow, which can be a potential risk to the cell function. Figure 7 illustrates that TAWSS decreases by an average of 25 and 30% at the downstream of entry tear in each case, respectively. According to Fig. 7a, TAWSS values along the aorta are decreased except in the outer surface of FL after the aortic arch (proximal FL) and some areas around DA. However, according to Fig. 6, in absolute terms (about 0.2 Pa), it is apparent that TAWSS does not differ significantly in these areas.

Oscillatory shear index (OSI) is another meaningful WSS indicator and can be achieved from the following equation^3^:

10 $$ OSI = \frac{1}{2}\left( {1 - \frac{{\left| {\frac{1}{T}\mathop \smallint \nolimits_{0}^{T} \vec{\tau }(t)dt} \right|}}{TAWSS}} \right) $$

**Figure 8:**
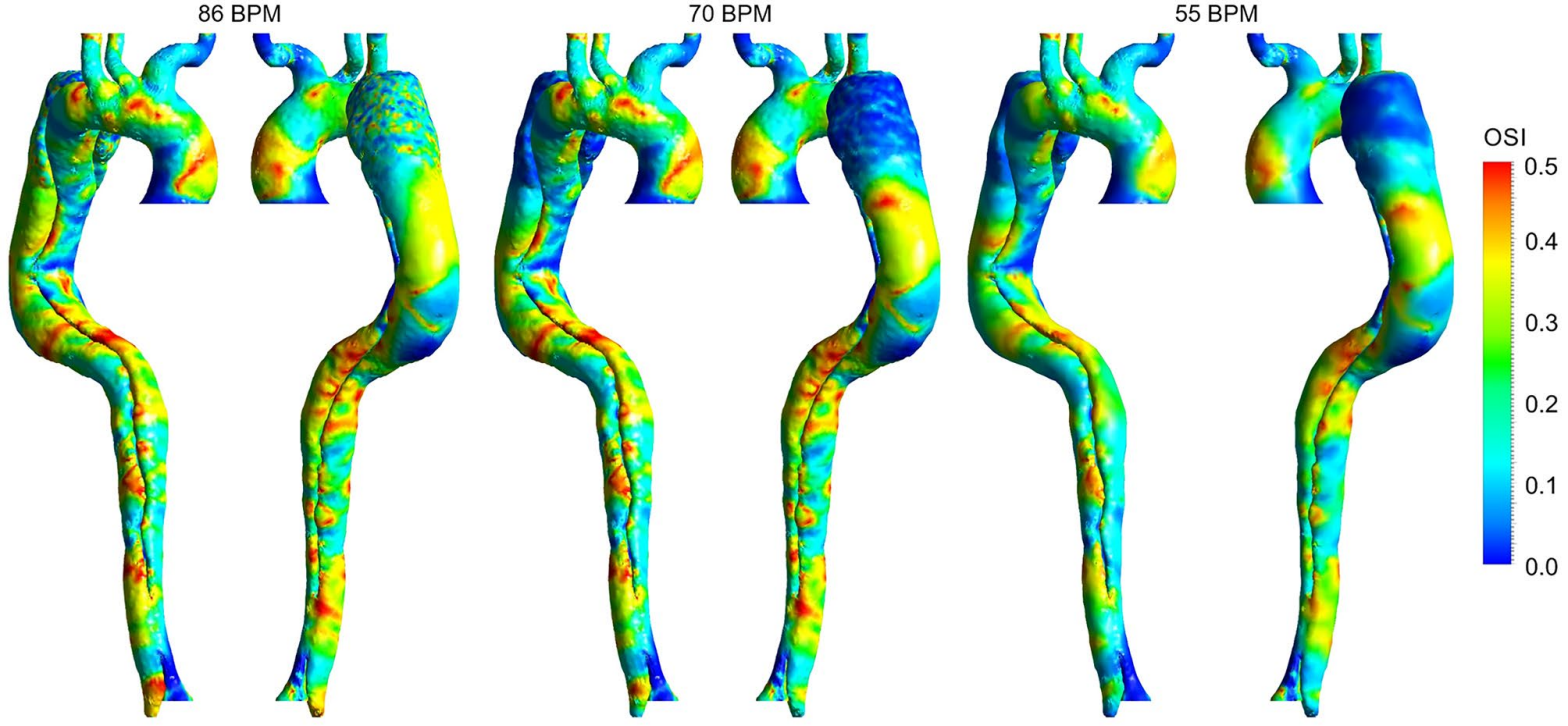
Oscillatory shear index (OSI) distribution for three cases (the scale bar shown in the figure indicates all contours).

**Figure 9:**
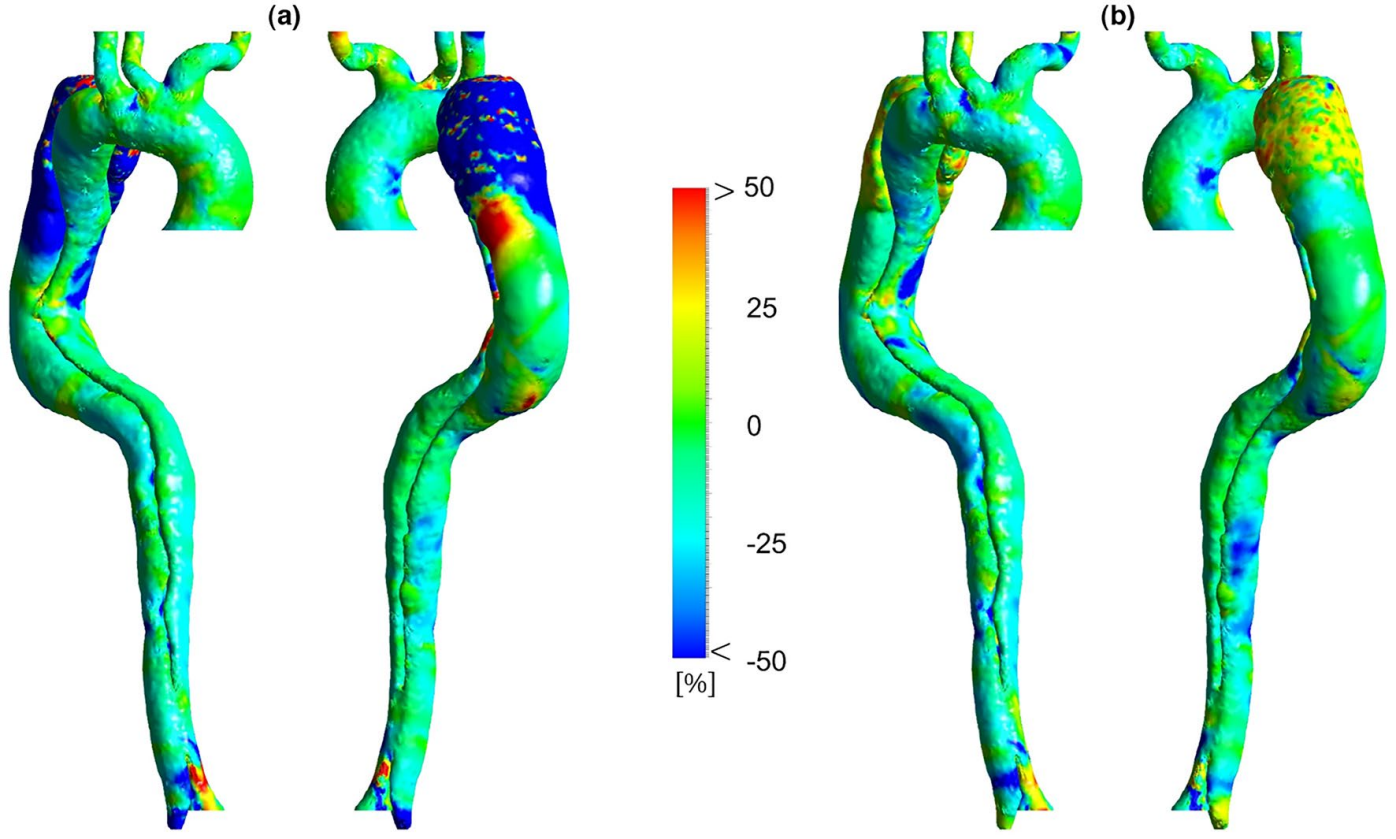
Percentage difference in oscillatory shear index (OSI) according to different heart rates. (**a**) Percentage difference in OSI between 86 and 70 BPM. (**b**) Percentage difference in OSI between 55 and 70 BPM.

This parameter measures the oscillation of forces on the endothelial cells^3^, which has a value between 0 and 0.5. A zero OSI quantity points to one-way wall shear forces, and the higher values indicate that the direction of WSS forces is rather unknown^3,4^. A similar sensitivity analysis for OSI at 86 and 70 BPM with different reductions in resistance values is performed, the results of which are shown in Supplementary Figures 1b and 2b. These results show the percentage difference in OSI throughout the domain varies between ± 2%. Thus, the OSI results in Figs. 8 and 9 are shown for the case with a 25% reduction in resistance at 86 BPM and the case with a 10% reduction in resistance at 70 BPM.

Figure 8 shows the OSI distribution for three cases. Generally, high OSI values are seen in the dispersed regions in the downstream entry tear, FL around DA, and some parts of the aortic arch due to the complicated and unstable flow. Figure 9 demonstrates OSI percentage differences according to different heart rates. OSI does not differ significantly between the models. However, by looking closely at some regions, some variations can be seen. The average percentage difference in OSI is approximately equal to ± 10% in most parts; however, some regions with a − 25% difference are found over the TL, indicating a reduction in the oscillating nature of the WSS forces. According to Fig. 9a, the highest percentage difference reduction in OSI is in the FL after the aortic arch and DA.

For a better understanding of the combination and interaction of these two characteristics (TAWSS and OSI), a parameter called HOLMES is investigated, given by^6^:

11 $$ HOLMES = TAWSS\;(0.5 - OSI) $$

**Figure 10:**
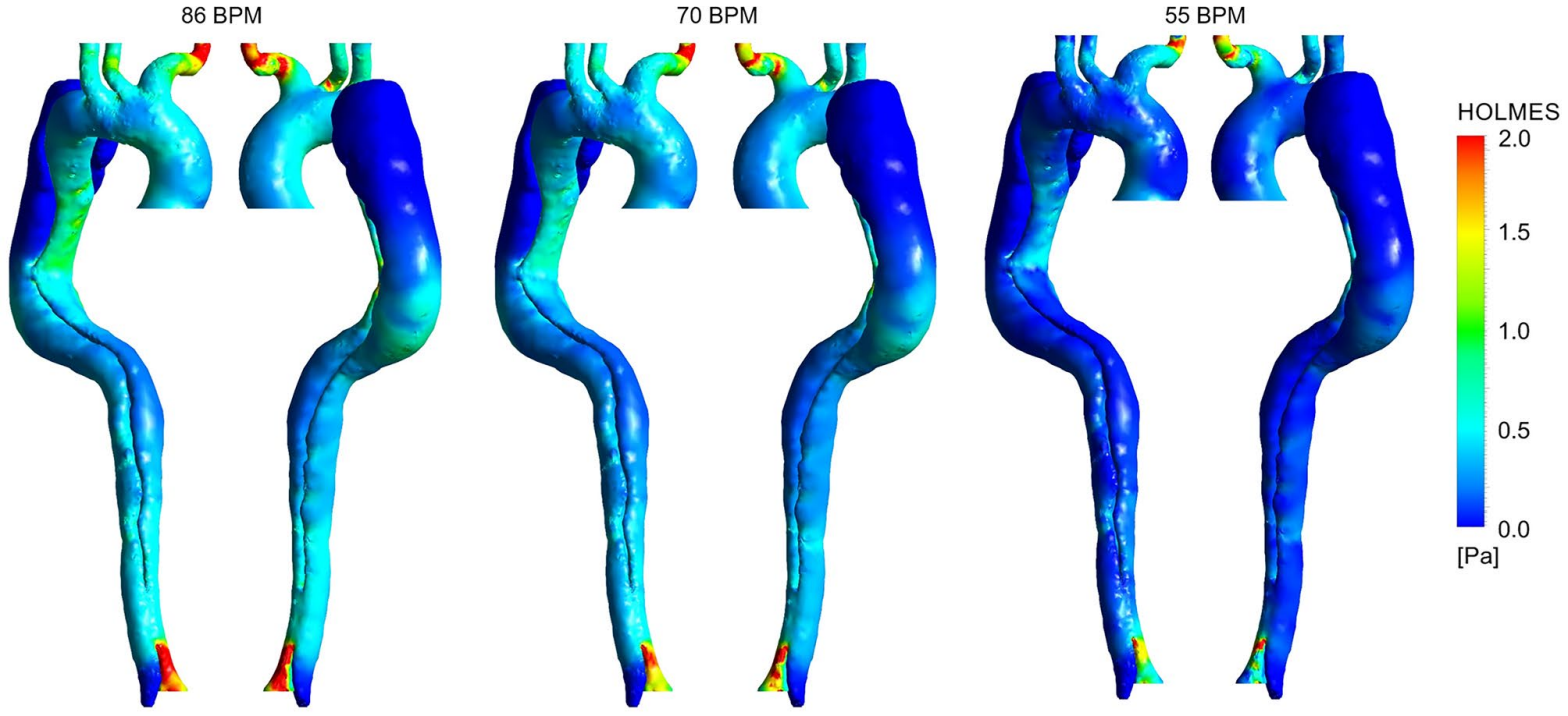
Highly oscillatory, low magnitude shear (HOLMES) distribution for three cases (the scale bar shown in the figure indicates all contours).

**Figure 11:**
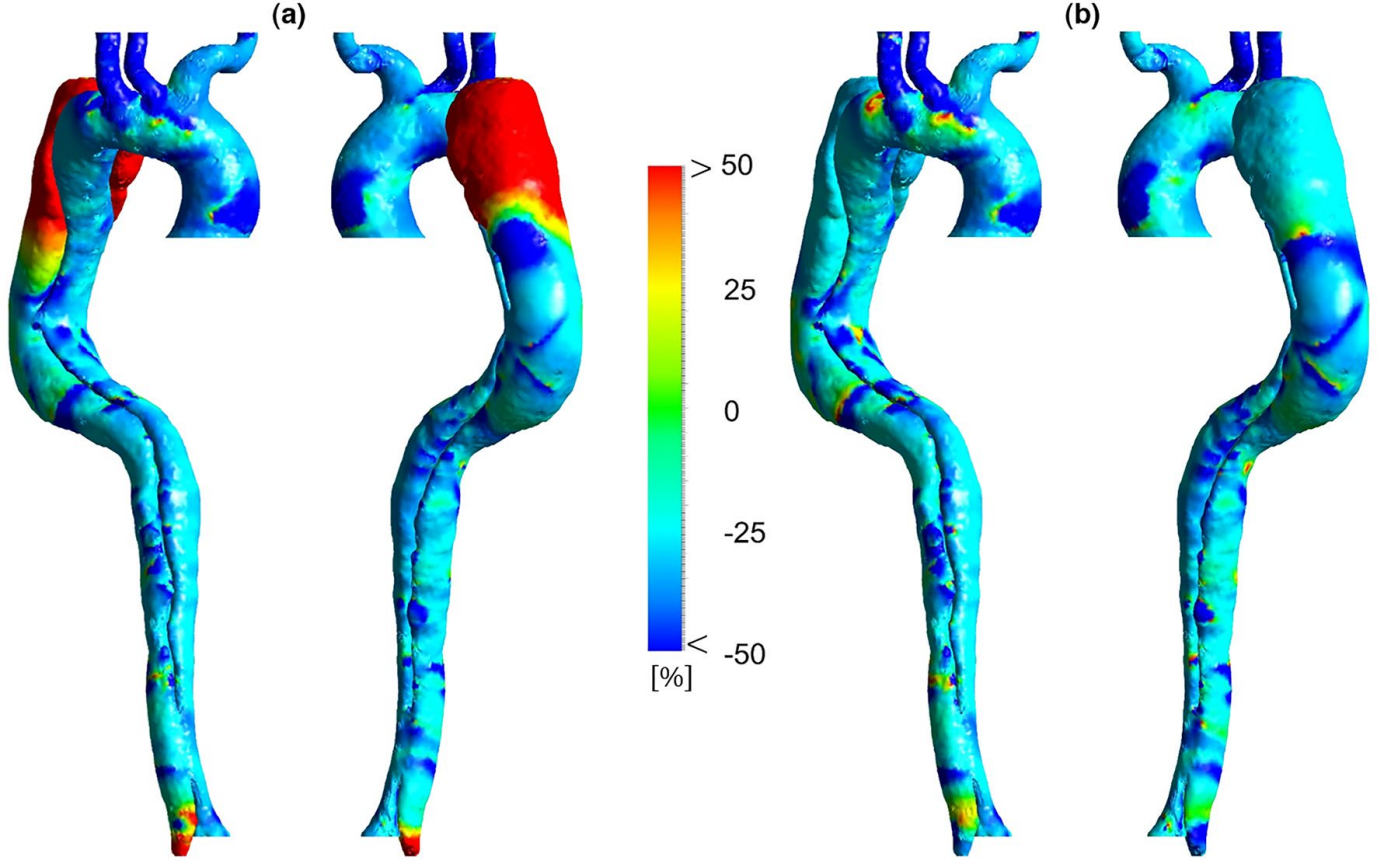
Percentage difference in highly oscillatory, low magnitude shear (HOLMES) according to different heart rates. (**a**) Percentage difference in HOLMES between 86 and 70 BPM. (**b**) Percentage difference in HOLMES between 55 and 70 BPM.

HOLMES provides an efficacious tool for predicting the location of plaques and their progression^6^. Both HOLMES distribution and its percentage difference between the three cases are shown in Figs. 10 and 11, respectively. The results of sensitivity analysis for HOLMES distribution at 86 and 70 BPM with different reductions in resistance values are shown in Supplementary Figures 1c, 2c, 5, and 6. According to Supplementary Figures 5 and 6, HOLMES distribution along the aorta at each heart rate remains approximately constant, where the percentage difference in HOLMES differs between ± 1% at most parts of the aorta (see Supplementary Figures 1c, 2c). Thus, the HOLMES distribution in Figs. 10 and 11 is shown only for the case with a 25% reduction in resistance at 86 BPM and the case with a 10% reduction in resistance at 70 BPM.

According to Fig. 10, the mean value of HOLMES is less than 1 Pa throughout the domain except for the upper branches (especially BT) and around the DA. According to Fig. 11, HOLMES is decreased along the aorta except in some scattered regions, i.e., FL after the aortic arch (proximal FL) and DA.

## Discussion

In the present study, results show significant hemodynamic differences between the three cases undergoing medical management. Analysis of the velocity changes in Fig. 3 provides evidence that flow resistance in the abdominal aorta in the moderate and regular-heart rate cases is decreased more than the high-heart rate one, which is a crucial issue during treatment in AD patients with malperfusion^6^. In studies of the development and progression of type-B AD, little attention has been paid to the role of complex flow around the tear. The most striking changes in Fig. 4 are the reducing distribution of some threatening patterns and high-velocity jet flows around the entry tear by successive heart rate reduction mechanism of BBs. These changes prevent the progression of FL, rupture of the vessel wall, and other pathologies, which are vital factors in patients treating medically^15^.

Pressure results in Fig. 5 indicate that the high-heart rate case suffers from an elevated mean pressure drop. The pressure drop during medical therapy in two other cases prevents severe co-occurring conditions in hypertension AD patients (i.e., calcification, extracellular fatty acid deposition, wall thickening, and fibrosis^4,14,15^). There is evidence showing that high-pressure FL particularly compresses the TL, notably during diastole, which can cause downstream organ ischemia or late aneurysmal dilatation^3,38^. The pressure results in the high-heart rate case demonstrate that high-pressure values in the FL are successfully decreased to the healthy range. What is interesting in pressure data is that during medical management, the average BP in the moderate and regular cases is decreased to lower values than a similar patient undergoing single-stenting and double-stenting operations in TEVAR, respectively^23^. These findings in this patient-specific paradigm are essentially helpful for supporting the clinical pre-procedural planning.

WSS is principally essential in the study of AD because of its direct effect on the progression and development of the vessel wall^3,4^, which cannot be measured invasively or experimentally. However, CFD can provide a great insight into this parameter and its indices^3,4,41^. For instance, high TAWSS values cause FL regions expand and grow, which increases the risk of rupture^3,6^, closing the TL, and completely losing function of the lower appendages^3,56^. TAWSS results represent that the high-heart case is suffered from abnormal hemodynamic stresses on the vessel wall around the entry tear and AA. Due to the lower velocity gradients in the moderate and regular models, TAWSS values in these acute areas are decreased to the healthy range (0–2 Pa^4,23^).

The variation of OSI in moderate and regular models turns into more stable, suggesting a better mechanical condition for the aortic endothelial cells. As reported by previous research^6,29,57^, an invaluable part of WSS indicators is that if an area exposes to a high OSI and low TAWSS, there is a high risk of rupture, calcification, or wall thickening. High OSI values in distal FL with low TAWSS (around DA) can potentially lead to high-risk complications. Due to the reduction of OSI values at these areas in both moderate and regular cases, possible adverse effects are controlled (see Fig. 9).

The results of follow-up mortality in acute type-B AD Patients have shown that a history of atherosclerosis causes a significant increase in the mortality rate^58^. Therefore, identifying the location of plaque formation and its progression during non-invasive treatment (using the shear stress index) can be of great help to clinicians. As discussed in the paper by Alimohammadi et al.^6^, the HOLMES index powerfully can predict regions to be prone to calcification with an accuracy of up to 95%. According to Fig. 11, interestingly, the increase in HOLMES at distal FL (around DA) regions ensures that plaque formation, calcification, and increasing endothelial cell permeability, which are subsequent common pathologies in type-B AD patients, are controlled successfully.

In conclusion, the present work is represented a personalised CFD simulation of blood flow in a patient suffering from type-B AD. The 3-D patient-specific geometry is coupled with dynamic boundary conditions and non-Newtonian viscosity blood flow to investigate and predict changes in hemodynamic metrics during virtual medical treatment. Results show the high-velocity jet and complex flow at entry tear, which both play a key role in AD patients treating pharmacologically, are managed in the moderate and regular cases. Additionally, the pressure results show that the high-risk elevated pressure drop in the case with high-heart rate successfully decreases with anti-impulse therapy. What is more, by decreasing the velocity gradient at the outer surface of TL around entry tear in the moderate and regular cases, TAWSS in these high-risk regions successfully decreases to a healthy range. Therefore, possible adverse effects such as the rupture of the vessel wall are controlled. The increase in HOLMES at distal FL demonstrates that plaque formation and calcification in this patient are managed. Overall, these investigated hemodynamic parameters not only can represent a great scheme to predict the patient’s conditions during medical treatment, but also play a vital role in planning the optimal treatment scenario for customised patient-specific therapy.

### Limitations

Although this study uses some patient-specific data; the geometry of type-B AD is reconstructed from CT images. Non-Newtonian blood viscosity and the tuned WK3 parameters from the previous study on a similar patient are used^51^, and a comprehensive sensitivity analysis on the resistance values of these parameters is performed; thus, the key hemodynamic outcomes predicted by CFD simulation is expected to be a suitable model of a patient undergoing virtual medical treatment.

The purpose of current work is to create a framework to further assist the clinicians with their patient-specific analysis. Thus, the inlet velocity profile is virtually adjusted based on the different heart rates during medical treatment. Furthermore, in the absence of personalised data; it is common to deploy similar data from the literature as used by^4,6,13,23,32,33,51,59^. However, the effect of the inlet velocity profile on the aortic hemodynamic is unsure^60^, and it is uncertain that parabolic or Womersley profiles provide any advancement over a uniform velocity^6,61,62^. Furthermore, building comprehensive models require patient-specific properties which are difficult to access in some cases due to a lack of advanced imaging facilities in hospital or patient’s conditions^60^. However, some research has found that through-plane (TP) inlet velocity profile can provide improved predictions of the hemodynamic results^60,63^ and in the absence of 4D-flow magnetic resonance imaging data adjusting the generic flow waveform based on the patient’s condition (stroke volume and heart rate) is the recommended solution^60^.

### Supplementary Information

Supplementary Information.

## Data Availability

All data used for this study are available from the author upon request.
