# Supplementary material for: The effect of beta-blockers on hemodynamic parameters in patient-specific blood flow simulations of type-B aortic dissection: a virtual study
Source: Sci Rep. 2021 Aug 6;11:16058. doi: 10.1038/s41598-021-95315-w (PMC8346572; doi:10.1038/s41598-021-95315-w)
Supplement: Supplementary file 1 — Supplementary Information. [file 41598_2021_95315_MOESM1_ESM.docx]

**The Effect of Beta-Blockers on Hemodynamic Parameters in Patient-Specific Blood Flow Simulations of Type-B Aortic Dissection: A Virtual Study**

**Mohammad Amin Abazari^1^, Deniz Rafiei^1^, M. Soltani^1,2,3,4,5,*^, Mona Alimohammadi^1,*^**

^1^ Department of Mechanical Engineering, K. N. Toosi University of Technology, Tehran, Iran

^2^ Department of Electrical and Computer Engineering, Faculty of Engineering, School of Optometry and Vision Science, Faculty of Science, University of Waterloo, Waterloo, Canada

^3^ Advanced Bio Initiative Center, Multidisciplinary International Complex, K. N. Toosi University of Technology, Tehran, Iran

^4^ Centre for Biotechnology and Bioengineering (CBB), University of Waterloo, Waterloo, ON, Canada

^5^ Cancer Biology Research Center, Cancer Institute of Iran, Tehran University of Medical Sciences, Tehran, Iran

^*^ Corresponding authors: msoltani@uwaterloo.ca and mona@alimohammadi.co.uk

Supplementary Table 1. Sensitivity analysis for blood pressure at 86 BPM with different resistance values.

| Parameter | Value | unit |
| --- | --- | --- |
| Average blood pressure at 86 BPM with a 25% reduction during pick systolic blood pressure along the aorta | 113.10 | mmHg |
| Average blood pressure at 86 BPM with a 20% reduction during pick systolic blood pressure along the aorta | 112.75 | mmHg |
| Percentage difference in blood pressure at 86 BPM with 25 and 20% reductions | 0.30 | _ |

Supplementary Table 2. Sensitivity analysis for blood pressure at 70 BPM with different resistance values.

| Parameter | Value | unit |
| --- | --- | --- |
| Average blood pressure at 70 BPM with a 15% reduction during pick systolic blood pressure along the aorta | 99.44 | mmHg |
| Average blood pressure at 70 BPM with a 10% reduction during pick systolic blood pressure along the aorta | 97.93 | mmHg |
| Percentage difference in blood pressure at 70 BPM with 15 and 10% reductions | 1.52 | _ |


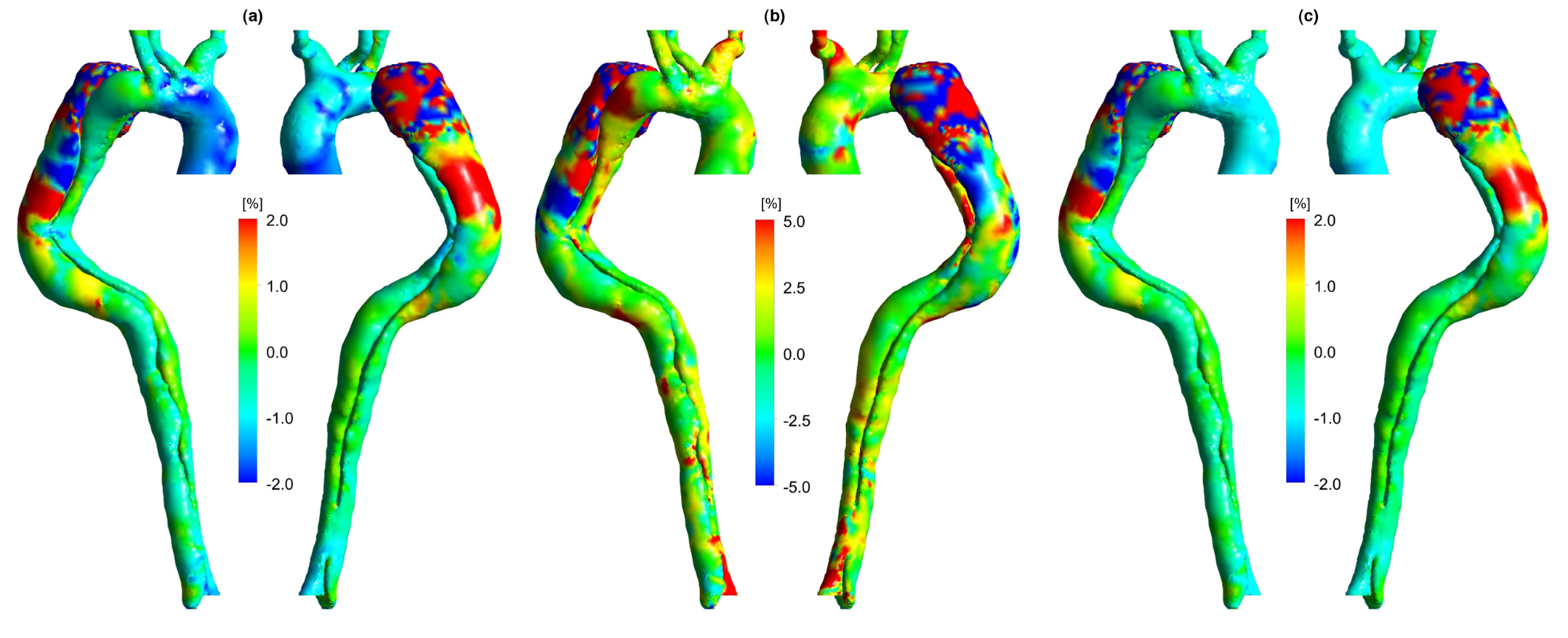


Supplementary figure 1. Sensitivity analysis for percentage difference in wall shear stress metrics at 86 BPM according to different resistance values. **(a)** Percentage difference in time-averaged wall shear stress between 25 and 20% reduction in resistance. **(b)** Percentage difference in oscillatory shear index between 25 and 20% reduction in resistance. **(c)** Percentage difference in highly oscillatory, low magnitude shear between 25 and 20% reduction in resistance.


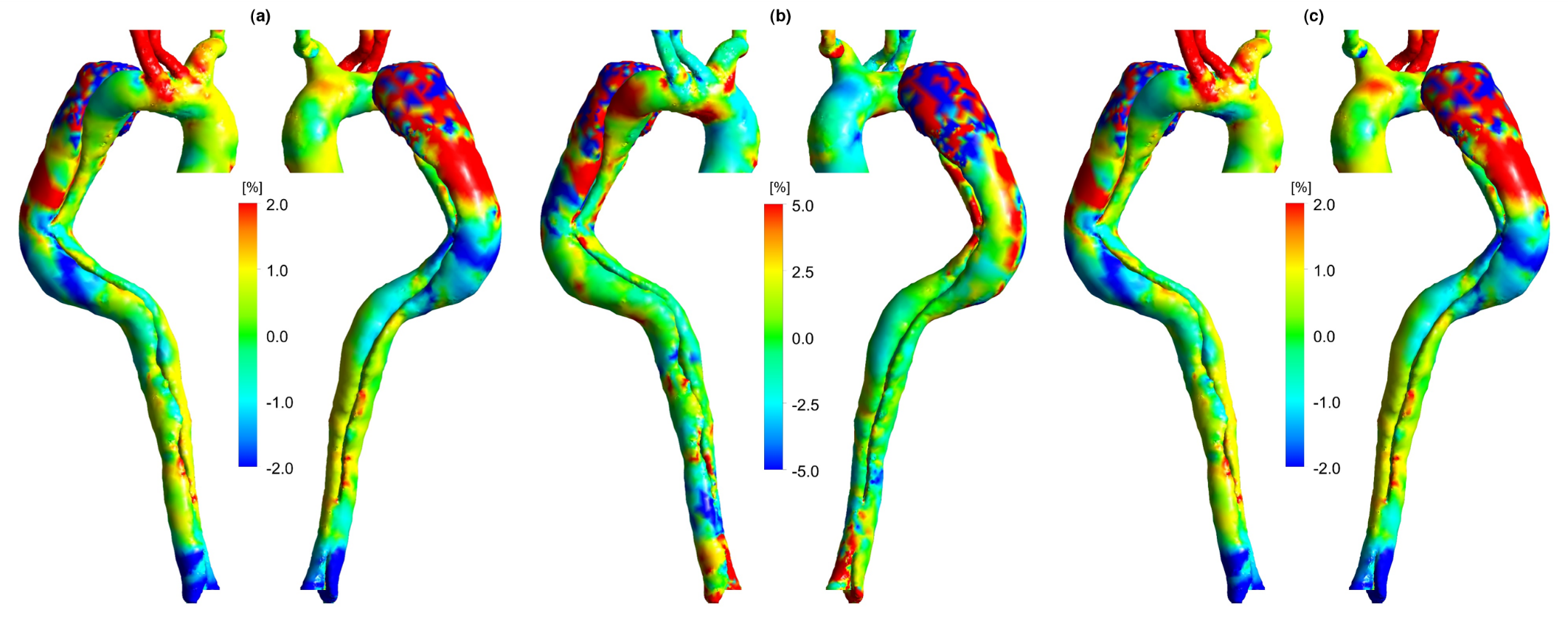


Supplementary figure 2. Sensitivity analysis for percentage difference in wall shear stress metrics at 70 BPM according to different resistance values. **(a)** Percentage difference in time-averaged wall shear stress between 15 and 10% reduction in resistance. **(b)** Percentage difference in oscillatory shear index between 15 and 10% reduction in resistance. **(c)** Percentage difference in highly oscillatory, low magnitude shear between 15 and 10% reduction in resistance.


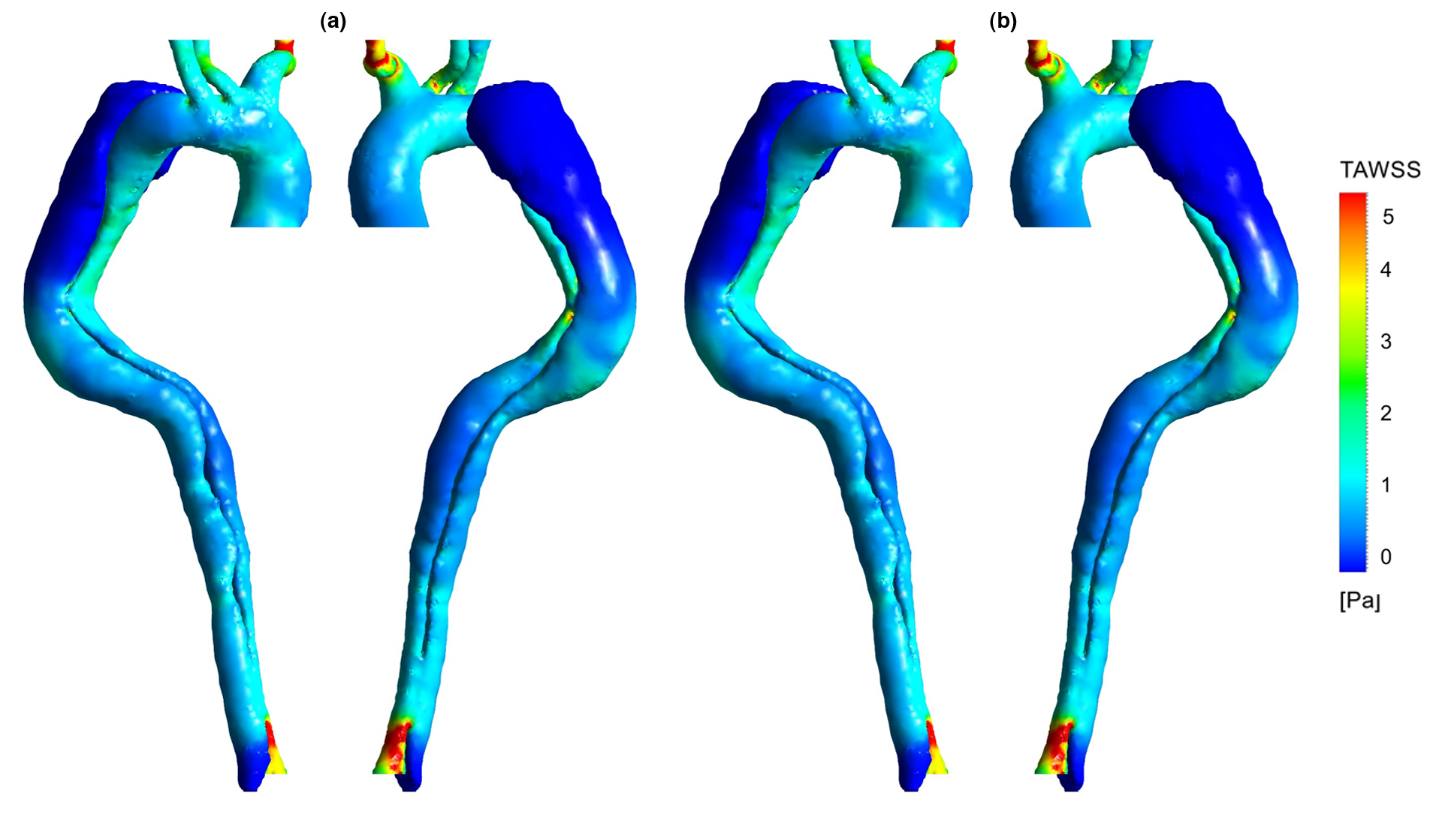


Supplementary figure 3. Sensitivity analysis for time-averaged wall shear stress (TAWSS) distribution at 86 BPM with different resistance values. **(a)** TAWSS distribution for a 20% reduction in resistance. **(b)** TAWSS distribution for a 25% reduction in resistance.


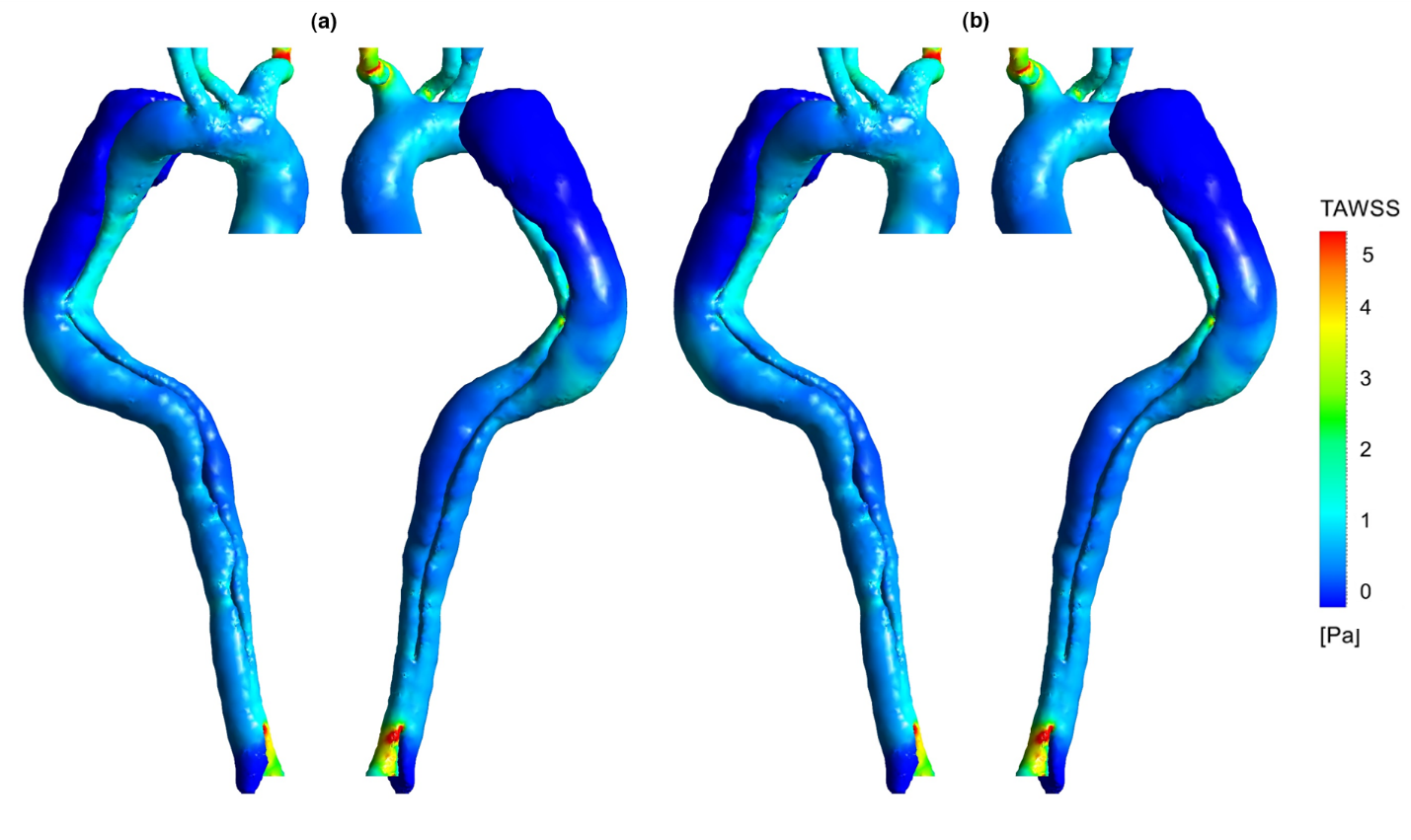


Supplementary figure 4. Sensitivity analysis for time-averaged wall shear stress (TAWSS) distribution at 70 BPM with different resistance values. **(a)** TAWSS distribution for a 15% reduction in resistance. **(b)** TAWSS distribution for a 10% reduction in resistance.


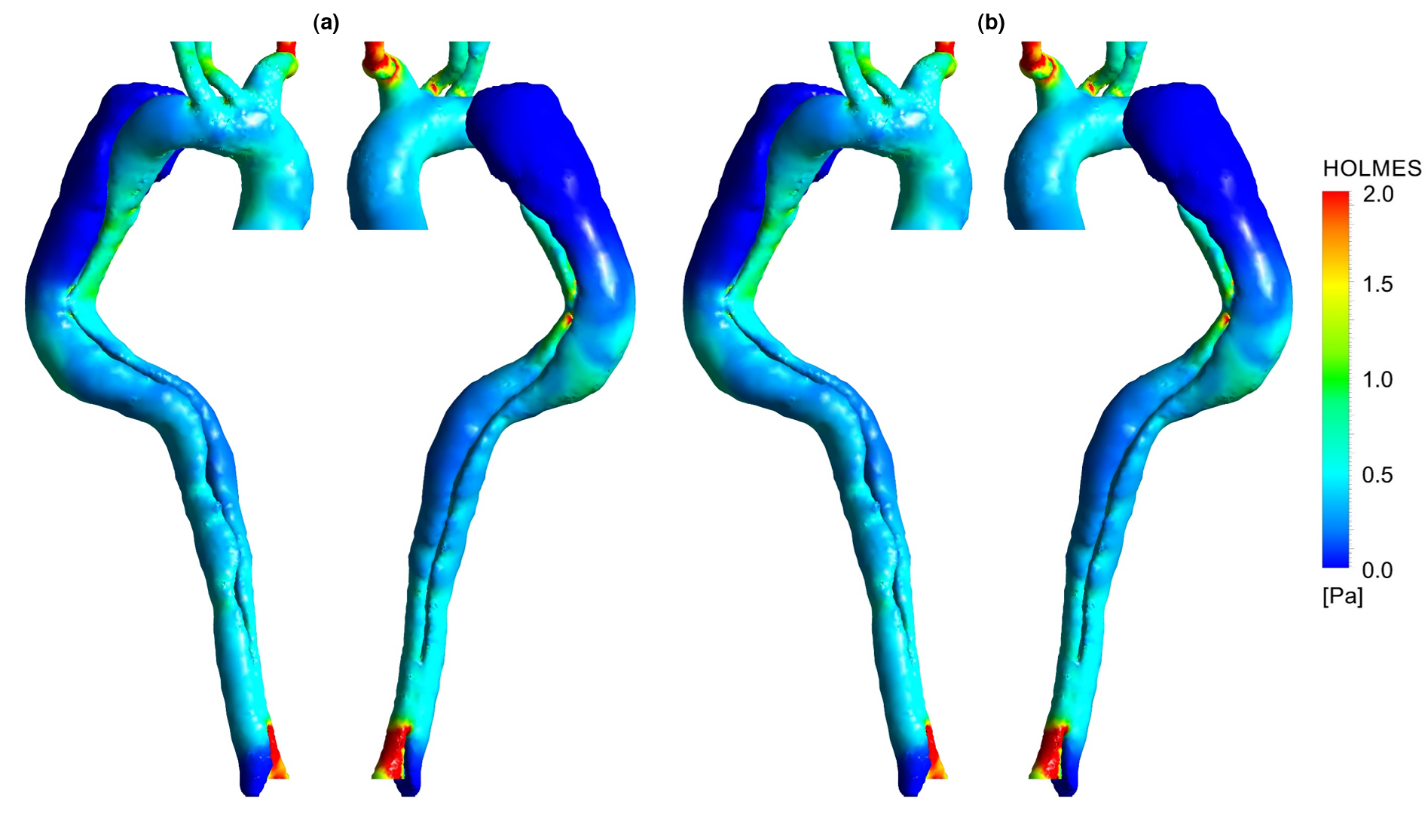


Supplementary figure 5. Sensitivity analysis for highly oscillatory, low magnitude shear (HOLMES) distribution at 86 BPM with different resistance values. **(a)** HOLMES distribution for a 20% reduction in resistance. **(b)** HOLMES distribution for a 25% reduction in resistance.


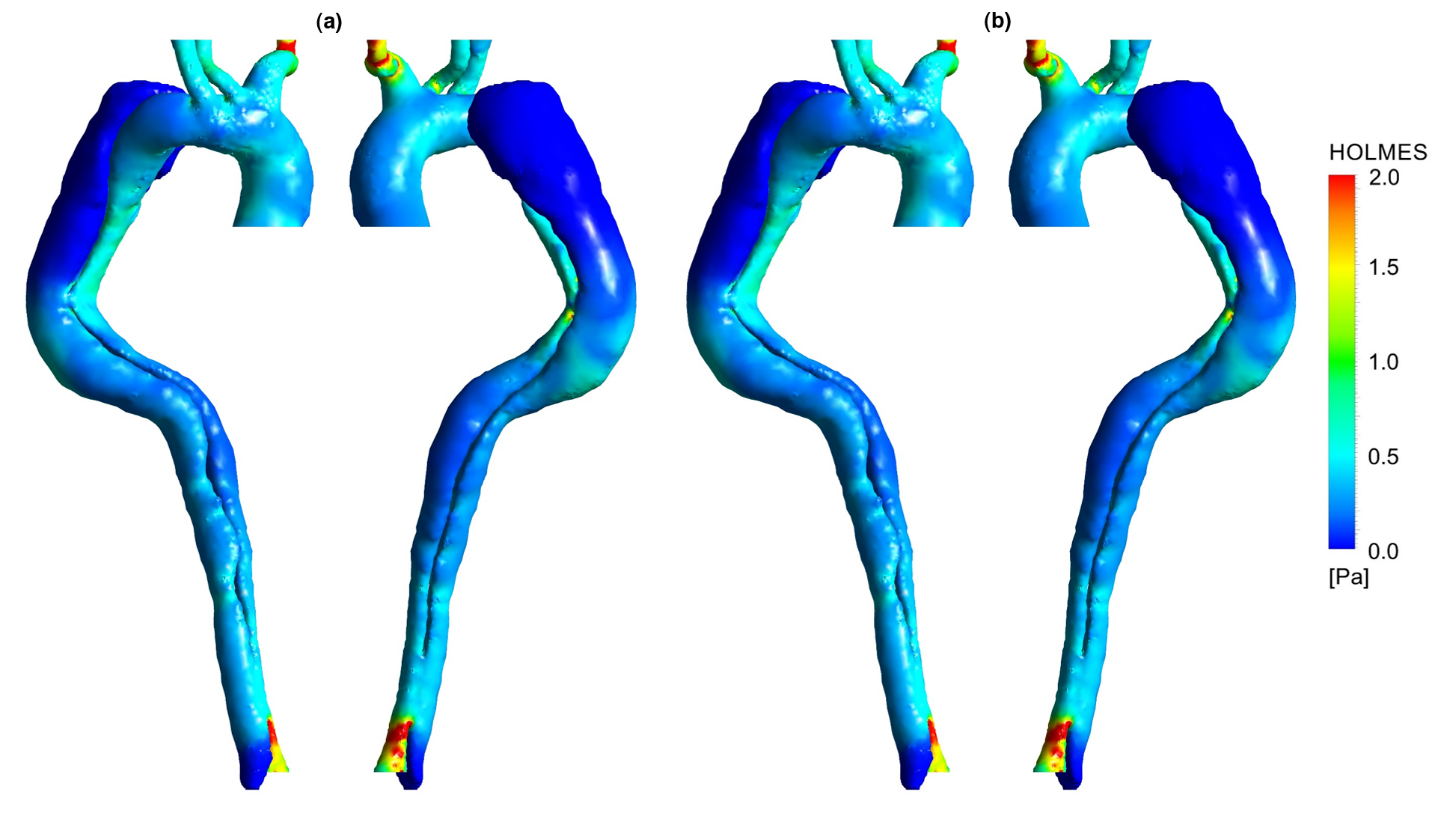


Supplementary figure 6. Sensitivity analysis for highly oscillatory, low magnitude shear (HOLMES) distribution at 70 BPM with different resistance values. **(a)** HOLMES distribution for a 10% reduction in resistance. **(b)** HOLMES distribution for a 15% reduction in resistance.
